# Shared Interoperable Clinical Decision Support Service for Drug-Allergy Interaction Checks: Implementation Study

**DOI:** 10.2196/40338

**Published:** 2022-11-10

**Authors:** Sungwon Jung, Sungchul Bae, Donghyeong Seong, Ock Hee Oh, Yoomi Kim, Byoung-Kee Yi

**Affiliations:** 1 Department of Health Sciences and Technology Samsung Advanced Institute for Health Sciences and Technology Sungkyunkwan University Seoul Republic of Korea; 2 Data Science Research Institute Samsung Medical Center Seoul Republic of Korea; 3 FirstDIS Ltd Seoul Republic of Korea; 4 Electronic Medical Records System Certification Criteria Development Department Korea Health Information Service Seoul Republic of Korea; 5 Department of Artificial Intelligent Convergence Kangwon National University Gangwon-do Republic of Korea

**Keywords:** clinical decision support, drug-allergy interaction, Health Level 7, Fast Healthcare Interoperability Resources, interoperability, CDS Hooks

## Abstract

**Background:**

Clinical decision support (CDS) can improve health care with respect to the quality of care, patient safety, efficiency, and effectiveness. Establishing a CDS system in a health care setting remains a challenge. A few hospitals have used self-developed in-house CDS systems or commercial CDS solutions. Since these in-house CDS systems tend to be tightly coupled with a specific electronic health record system, the functionality and knowledge base are not easily shareable. A shared interoperable CDS system facilitates the sharing of the knowledge base and extension of CDS services.

**Objective:**

The study focuses on developing and deploying the national CDS service for the drug-allergy interaction (DAI) check for health care providers in Korea that need to introduce the service but lack the budget and expertise.

**Methods:**

To provide the shared interoperable CDS service, we designed and implemented the system based on the CDS Hooks specification and Health Level Seven (HL7) Fast Healthcare Interoperability Resources (FHIR) standard. The study describes the CDS development process. The system development went through requirement analysis, design, implementation, and deployment. In particular, the concept architecture was designed based on the CDS Hooks structure. The MedicationRequest and AllergyIntolerance resources were profiled to exchange data using the FHIR standard. The discovery and DAI check application programming interfaces and rule engine were developed.

**Results:**

The CDS service was deployed on G-Cloud, a government cloud service. In March 2021, the CDS service was launched, and 67 health care providers participated in the CDS service. The health care providers participated in the service with 1,008,357 DAI checks for 114,694 patients, of which 33,054 (3.32%) cases resulted in a “warning.”

**Conclusions:**

Korea’s Ministry of Health and Welfare has been trying to build an HL7 FHIR-based ecosystem in Korea. As one of these efforts, the CDS service initiative has been conducted. To promote the rapid adoption of the HL7 FHIR standard, it is necessary to accelerate practical service development and to appeal to policy makers regarding the benefits of FHIR standardization. With the development of various case-specific implementation guides using the Korea Core implementation guide, the FHIR standards will be distributed nationwide, and more shared interoperable health care services will be introduced in Korea.

## Introduction

Clinical decision support (CDS) can improve health care with respect to the quality of care, patient safety, efficiency, and effectiveness [1,2]. In addition, it can reduce the cognitive burden of the physicians upon using the order sets such as procedures and prescriptions [3]. In combination with electronic health records (EHRs), the CDS system influences the behavior of physicians and increases adherence to clinical guidelines [1,4].

However, adopting a CDS system in a health care setting remains a challenge [4,5]. Some hospitals have used in-house developed CDS systems or commercial CDS solutions [6]. Since an in-house CDS system tends to be tightly coupled with a specific EHR system, the functionalities and knowledge base are not easily sharable. On the other hand, a commercial CDS requires costly integration with existing EHR systems both in terms of time and effort. The situations are worse with small- to medium-sized hospitals, including clinics. Lack of budget and expertise prevents them from implementing CDS services [7,8].

Shared interoperable CDS services that enable sharing the knowledge base and expansion of the CDS service can mitigate the previous problems. The services can be implemented using the CDS Hooks, that is, Health Level Seven (HL7) International–published specifications for CDS [9]. The CDS Hooks provides a way to call external CDS services remotely within a provider’s workflow [10]. It also uses the HL7 Fast Healthcare Interoperability Resources (FHIR) as a data model. By using the FHIR, the CDS services can provide interoperability to health care providers: tertiary hospitals, small- to medium-sized hospitals, and clinics operating on heterogeneous EHR systems. The result of the decision support is to return the cards displaying text, suggestions, or links to launch a Substitutable Medical Applications, Reusable Technologies (SMART) application [11-14].

Since 2011, the Health Insurance Review and Assessment Service (HIRA) in Korea has provided the drug utilization review (DUR) program as a CDS system containing real-time drug safety data for doctors and pharmacists. The DUR program presents 11 review items, including drug-drug interactions, duplicate prescriptions, and drug regimen dose and duration. The DUR system has been distributed among over 99.8% of health care providers as of 2019 [15-18]. Nonetheless, the adoption of other available CDS services remains a challenge.

Korea’s Ministry of Health and Welfare (MoHW) oversees several national initiatives to apply and distribute interoperable health IT standards. As one of several national initiatives, feasibility studies are ongoing to embrace the HL7 FHIR standards [19], widely adopted in the global health care industry [20].

In this study, we focus on developing and deploying the sharable and interoperable CDS service for the drug-allergy interaction (DAI) check based on the CDS Hooks specification at the national level. The main objective of CDS service in the initial stage is technical feasibility and service availability. The DUR program in Korea does not cover the DAI check due to low awareness of the social burden and its prevention for the DAI when setting the review items in 2011 [21]. Global concerns regarding DAI are increasing, and inappropriate medication prescriptions frequently occur in all health care settings [22,23]. Implementation of CDS service for the DAI check is relatively more accessible than other CDS services [12]. The HL7 FHIR standard and CDS Hooks specification allow the CDS service to be sharable, interoperable, and scalable. The study is expected to be a starting point for the national adoption of the HL7 FHIR standards.

## Methods

### Overview

We developed a shared interoperable CDS system based on CDS Hooks for a DAI check to provide a service to health care providers. The system is triggered by medication orders in the EHR system. When it is evoked, the system checks the DAI and returns recommendations back to the provider. We developed the system in the following steps: (1) requirement analysis, (2) design, (3) implementation, and (4) deployment. In the first step, we identified data elements used for DAI check and classified them into mandatory and optional. We also selected the FHIR resources for contextual information available within an EHR system. Second, we designed concept architecture and web service end points, representational state transfer (RESTful) application programming interfaces (APIs), based on the CDS Hooks structure. We profiled FHIR resources according to data elements and specified the card, a form that represented a result of decision support. We designed a rule engine including a four-step drug-allergy screening logic and knowledge base. Lastly, we implemented components and functions, and deployed the CDS system on a government-managed cloud service called G-Cloud.

The CDS service can simultaneously be used by multiple health care providers, such as tertiary hospitals, small- to medium-sized hospitals, and clinics with their own EHR systems. Health care providers can DAI check using a remote CDS service call when ordering medications. An EHR system creates a request payload with patients’ prescriptions and allergy data, and transmits it to the CDS service. The CDS service executes the DAI check logic using the request payload and then returns the result to the EHR system.

### Concept Architecture

**Figure 1:**
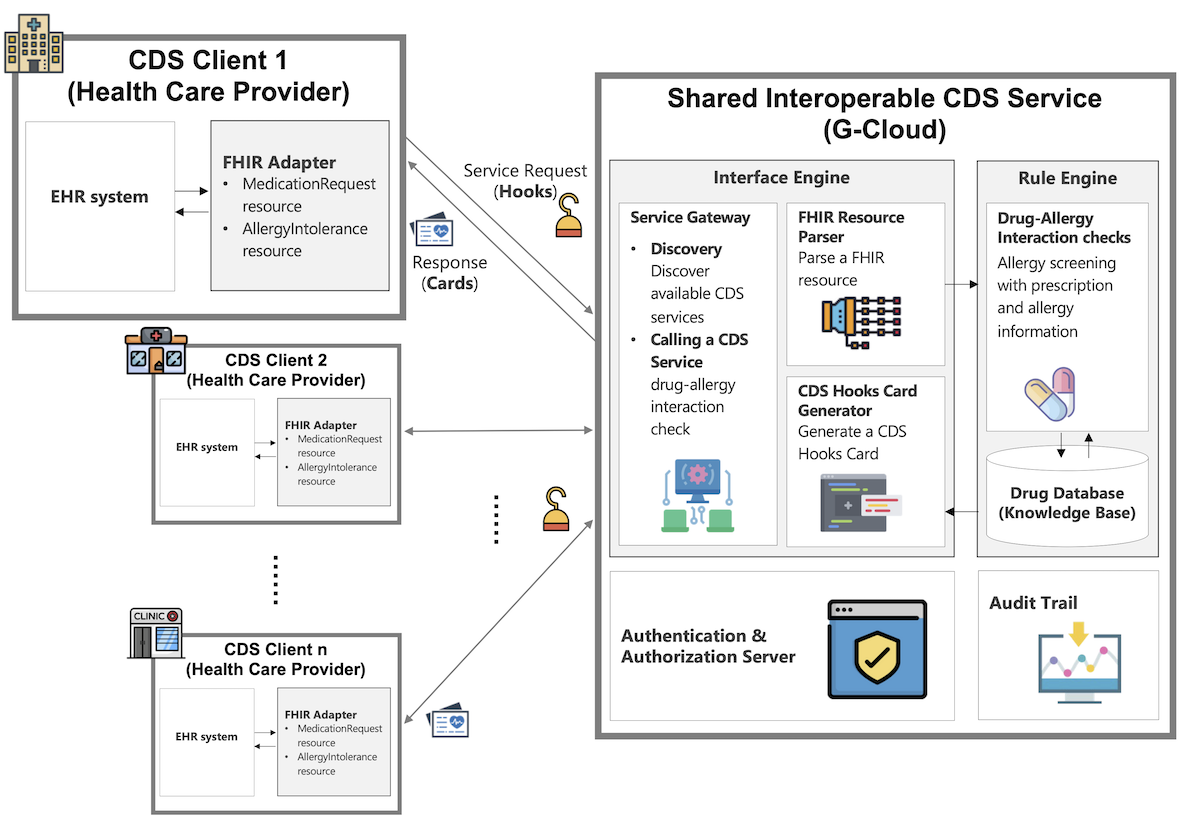
The concept architecture for the shared interoperable CDS system is based on CDS Hooks anatomy. Multiple health care providers simultaneously invoke the shared interoperable CDS service deployed on G-Cloud using a hook and receive a card as a response. CDS: clinical decision support; EHR: electronic health record; FHIR: Fast Healthcare Interoperability Resources.

We designed a concept architecture according to the CDS Hooks structure, which consists of CDS services, CDS clients, and cards, as shown in Figure 1. The CDS clients that are EHR systems in health care providers invoke the CDS service through a hook that is an event trigger, and the CDS service provides recommendations using a card to the CDS clients. The CDS system was implemented in version 1.0 of the CDS Hooks specification.

The CDS service was designed as a cloud service and consisted of an interface engine, rule engine, authentication and authorization server, and audit trail. The interface engine has three components: the service gateway, the FHIR resource parser, and the CDS Hooks card generator. The service gateway provides a discovery end point and DAI check end point, and the FHIR resource parser parses request payload data to relay to the rule engine. The CDS Hooks card generator creates decision support results as a card to return to the CDS client. The rule engine checks the DAI using the prescription and patients’ allergy information and then returns a result of allergy screening to the CDS Hooks card generator. The authenticate and authorization server authenticates the EHR system using an issued token, and the audit trail monitors which health care providers invoke the service and when and how often they use it.

We applied the CDS Hooks security model with some variations to the CDS service. The CDS Hooks specification provides a security model, such as mutual identification, transport layer security protocol, and JSON web token. We developed the authentication and authorization server to provide a token to CDS clients. The token issued by the CDS service authenticates the CDS client. It reduces the burden on the health care provider’s authentication server development and helps a wider adoption of the CDS service. In addition, a whitelist of health care providers is managed based on our risk management strategy.

The CDS client creates an HTTP request to the CDS Hooks service with parameters that include required fields (hook, hookInstance, and context) and optional fields (fhirServer, fhirAuthorization, and prefetch). The context and prefetch fields have the FHIR resources, which are translated by the FHIR adapter. The FHIR adapter was considered instead of an FHIR server since the adoption of the FHIR standard is in its infancy in Korea.

### FHIR Resources Profile

**Figure 2:**
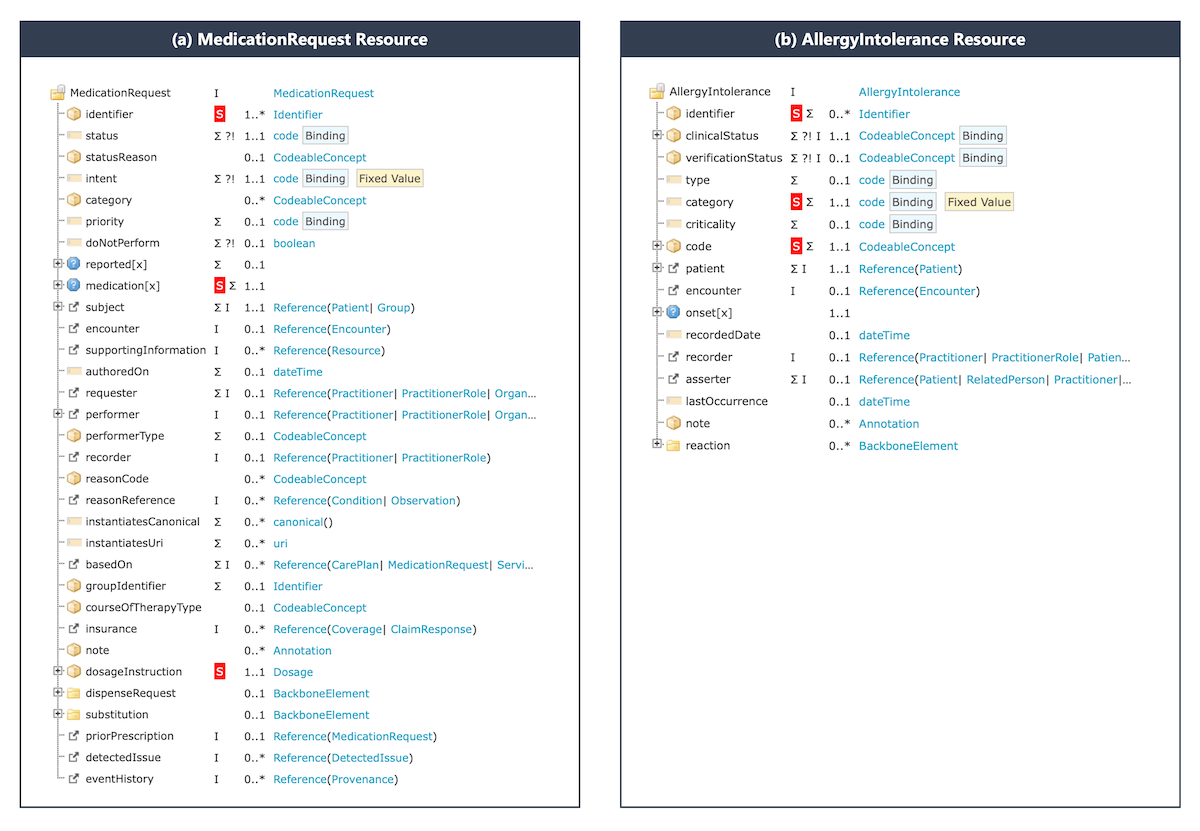
The MedicationRequest and AllergyIntolerance resource profile. The resources profiled for the clinical decision support service are inherited from the Korea Core Implementation Guide 1.0.0. Elements with "must support" are marked with an "S" in the red square.

We identified data elements for the DAI check and profiled two FHIR resources, MedicationRequest and AllergyIntolerance, based on the FHIR R4 (v4.0.1) [24] and Korea (KR) Core implementation guide (IG) v1.0.0-STU 1 [25]. The MedicationRequest resource represents a supply of the medication and administration instructions, as shown in Figure 2A [24]. There are two options for representing medication information in the MedicationRequest resource: referencing the Medication resource to the medicationReference element and assigning the medication code directly to the medicationCodeableConcept element. In this profile, we applied the latter because health care providers do not manage the Medication resource. The medicationCodeableConcept element is bound to the Korea Drug (KD) code, the national code system to identify and manage drug products [26]. The cardinality and must support constraints of the MedicationRequest resource are inherited by the KR Core MedicationRequest Profile. The CDS service uses the medication element but not the identifier nor the dosageInstruction elements, although they are marked as must support. Elements designated as a must support are necessary conditions for the FHIR resource to be exchanged, but consumers of the resource do not necessarily have to use all must support elements in principle.

The AllergyIntolerance resource represents a record of a clinical assessment of an allergy or intolerance, as shown in Figure 2B [24]. The category element with the AllergyIntoleranceCategory value set is assigned the fixed value of “medication.” The code element is bound to a proprietary value set developed by the vendor that provides the rule engine of the CDS service, since there is no national code system that identifies the allergy or intolerance.

The cardinality constraints of the AllergyIntolerance resource are inherited by the KR Core IG. The identifier, category, and code elements are marked as “must support.” The profiled resources are published to SIMPLIFIER.NET, one of the FHIR registries.

**Figure 3:**
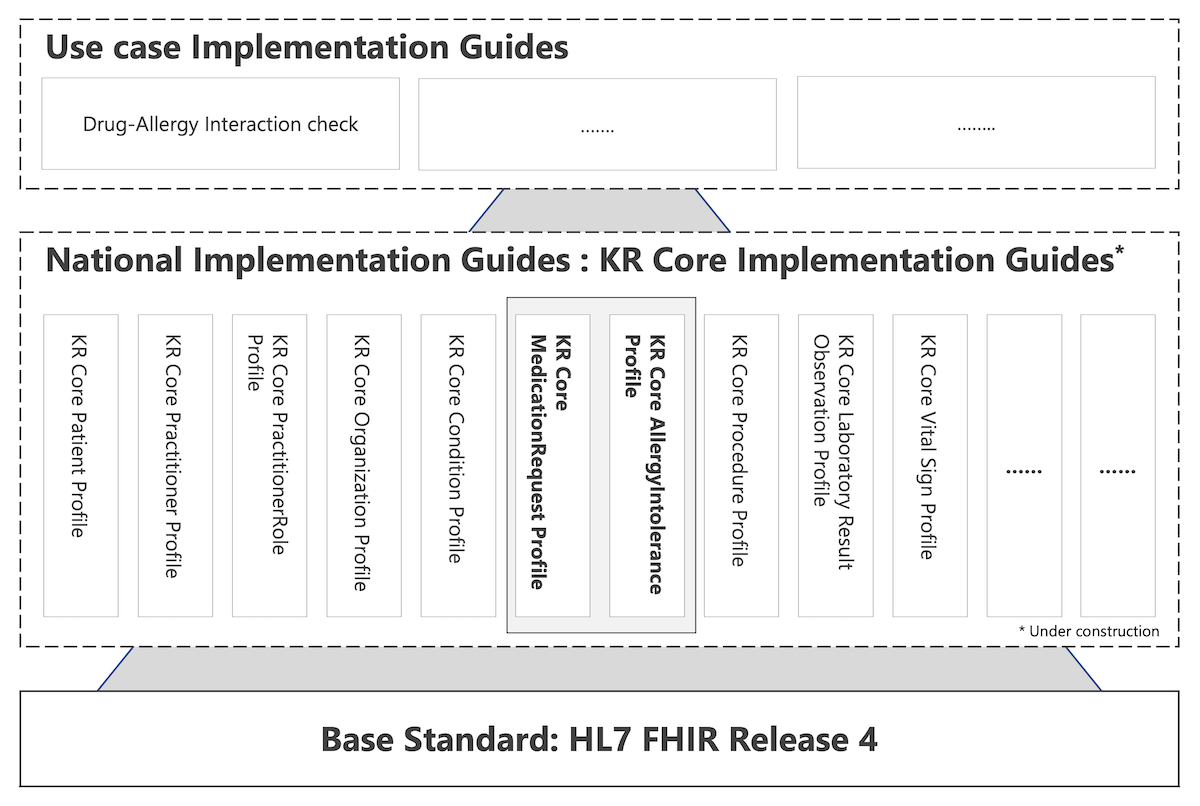
The MedicationRequest and AllergyIntolerance resources profiled through the shared interoperable clinical decision support system are conformed with the KR Core MedicationRequest profile and KR Core AllergyIntolerance profile. FHIR: Fast Healthcare Interoperability; HL7: Health Level Seven; KR: Korea.

Two profiled resources are conformant to the KR Core IG in Figure 3. The KR Core IG, a national-level FHIR IG, such as the US Core [27], UK Core [28], Australian Base [29], and Canadian Baseline [30], is essential in the nationwide adoption of the FHIR standards and in building an ecosystem based on the standards. We expect that specific use case FHIR IG based on the resource profiles proposed in this study will be adopted as a national standard in Korea.

### CDS Service Interfaces and Cards

**Figure 4:**
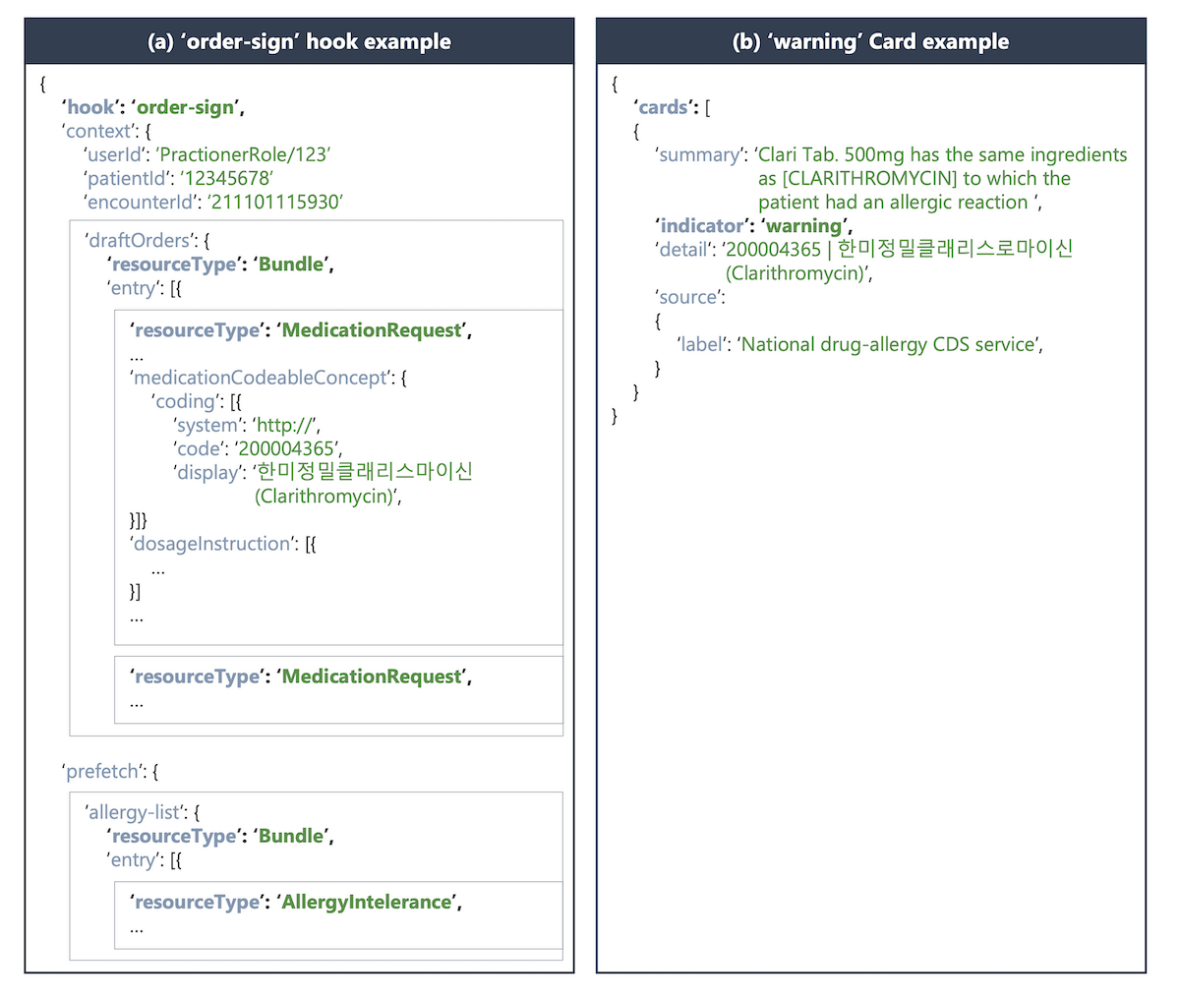
Examples of order-sign hook and warning card. The order-sign hook has userId, patientId, and draftOrders as required fields, but userId is not used in the clinical decision support (CDS) service for the drug-allergy interaction (DAI) check. The card, the response of the CDS service, includes the results of the DAI check, suggested actions, and links to the launch app.

Two end points were designed and implemented: the discovery and DAI check APIs. The discovery API provides the list of CDS services, including a description of the CDS service and any requested data to be prefetched [9]. The DAI check API is the CDS service using the “order-sign” hook, as shown in Figure 4A. The order-sign hook occurs when the provider is ready to sign one or more orders for a patient, and it has the userId, patientId, and draftOrders as required fields and encounterId as optional. The userId field is included since it is required for the order-sign hook and not used for any other purposes. The CDS service does not distinguish individual providers invoking the service since it does not require a physician ID for DAI checks. The draftOrders field has a Bundle resource that lists MedicationRequest resources. The AllergyIntolerance resources are attached in the prefetch field that describes the relevant data required in the CDS service.

The CDS service responds to the CDS client with cards containing information, suggested actions, and links to launch an application. The DAI check API returns a card with a “warning” indicator, as shown in Figure 4B. The cards are JSON documents and have several fields, such as summary, indicator, and source field. The summary field is a summary message for display to the provider, and the importance of the card is represented by the following indicators: “info,” “warning,” and “critical.” The source field is a source of information displayed on this card.

### Rule Engine

**Figure 5:**
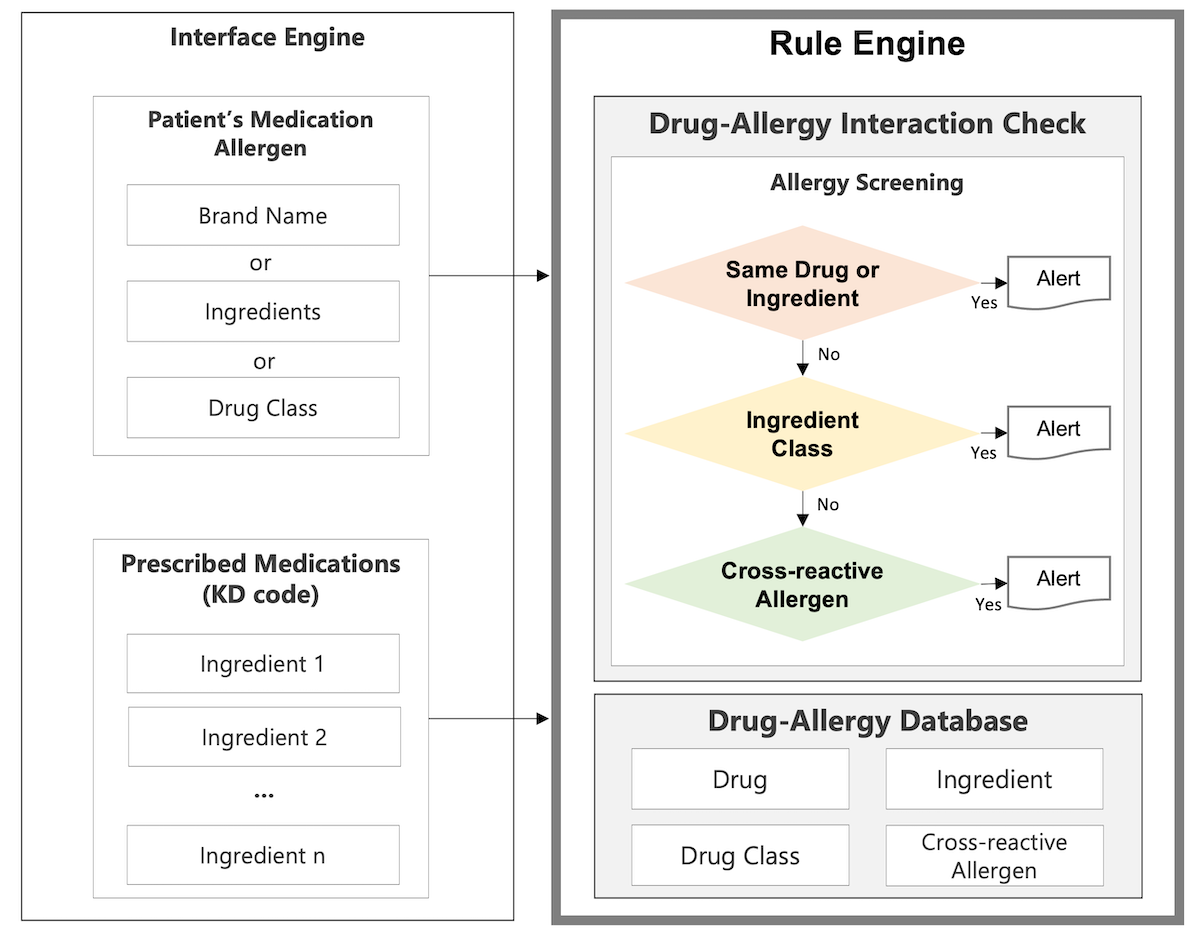
The three-step drug-allergy interaction check screening process: (1) check whether allergens and prescribed medications have the same product or ingredient, (2) check whether they belong to the same drug or ingredient class, and (3) check whether they have cross-reactive allergens. KD: Korea Drug.

We designed and developed the rule engine to check an interaction between a patient’s medication allergens and prescribed medications transmitted from a health care provider, as shown in Figure 5. The allergen data from the AllergyIntolerance resource can be a brand name, substance, or drug class. The medication data from the MedicationRequest resource is a brand name coded by the KD code managed by the HIRA. The DAI check is performed in a three-step screening process: (1) check whether allergens and prescribed medications have the same product or ingredient, (2) check whether they belong to the ingredient class, and (3) check whether they have a cross-reactive allergen.

The Drug Allergy database consists of master and relation tables. The master tables are the Drug, Drug Class, Ingredient, and Cross-Reactive Allergen. The Drug and Drug Class tables uniquely identify regulated medicinal products using the KD code as the primary key. The Ingredient table models substances that constitute a medicinal product and includes columns such as ingredient code, name, and synonym. These tables are designed based on the Identification of Medicinal Products, a suite of five International Organization for Standardization standards to facilitate the reliable exchange of medicinal product information. The Ingredient table is related to the Cross-Reactive, Drug, and Drug Class tables by each primary key. In addition to these tables, the drug allergy database has several relation tables used to perform DAI checks.

### Ethical Considerations

This study did not require ethics approval as no personal data was collected, and no interventions were implemented.

## Results

In this study, the national CDS service for the DAI check was developed to ensure the safe use of medicine and was deployed on G-Cloud, a government cloud service established and run by National Computing and Information Service in Korea [31]. The CDS service was launched in March 2021 and has been operated by the Korea Health Information Service. As shown in Table 1, a total of 67 providers participated in the service with 1,008,357 DAI checks for 114,694 patients, of which 33,054 (3.32%) resulted in a “warning” [32]. The results were obtained by analyzing the log data accumulated in the audit trail system.

**Figure 6:**
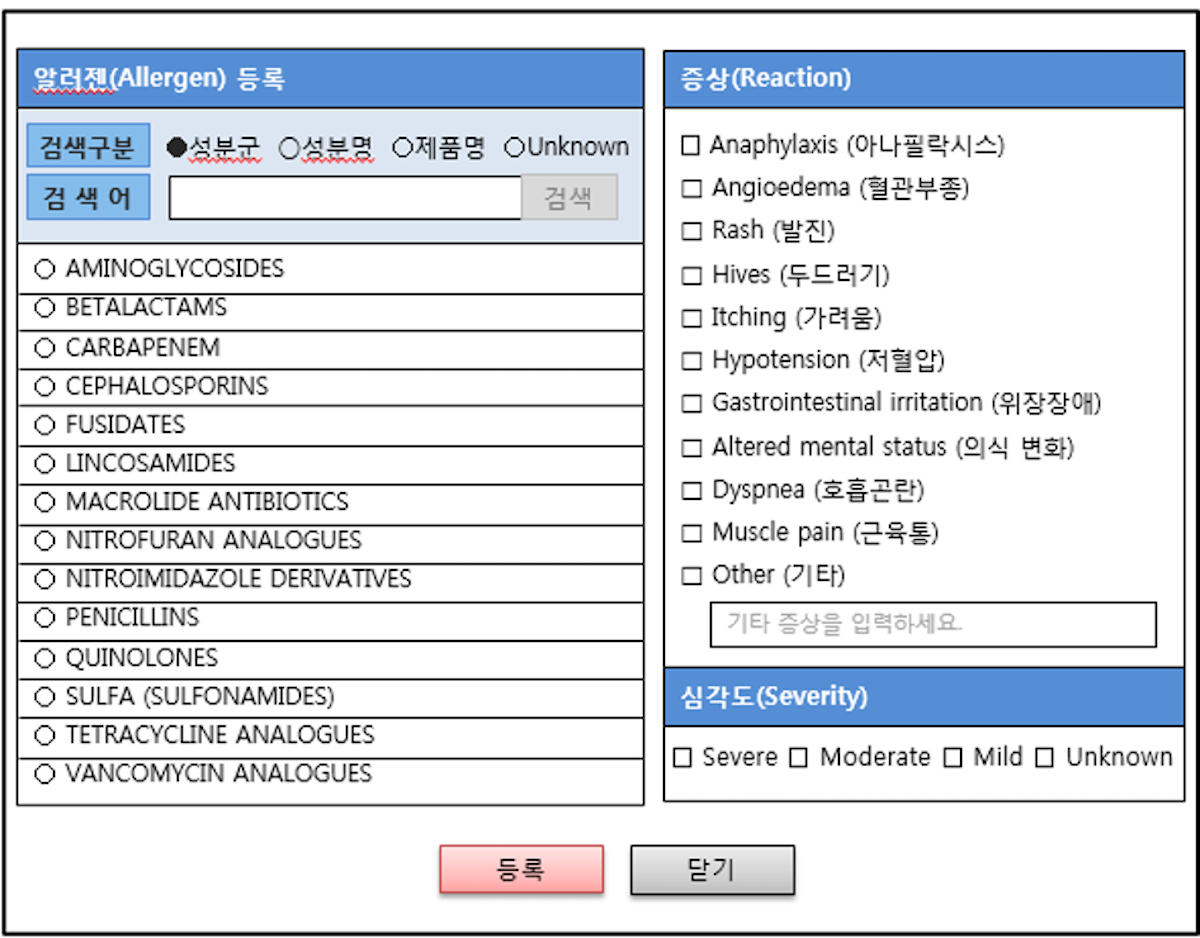
Screenshot of the reference implementation for a patient’s allergen inquiry. To drug-allergy interaction check, physicians should retrieve a patient's allergen code through reference implementation provided by the clincal decision support service.

Physicians use the national CDS service for the DAI checks when prescribing medications. The physicians should search the allergen codes provided by the CDS service before calling the CDS service. Since Korea does not yet have a national standard allergy code system, most health care providers store allergy data for the patient as text. To use the CDS service, physicians are also expected to search for an allergy code in the proprietary value set. For this extra step, the CDS service IG provides a reference implementation to inquire about the allergen, allergic reaction, and severity codes, as shown in Figure 6. The health care providers or EHR vendors are expected to develop the component and integrate it with their EHR systems.

**Table 1:** Results of the shared interoperable CDS service for drug-allergy interaction check in December 2021.

Result category		Amount
**Participants, n**		
	Health care providers	67
	Patients	114,694
**CDS^a^ service requests, n**		
	Drug-allergy interaction checks	1,008,357
**CDS service responses, n (%)**		
	Warning cards	33,504 (3.32)
	No responses	974,853 (96.68)

^a^CDS: clinical decision support.

## Discussion

### Principal Results

The study applied the CDS Hooks specification to provide the nationwide shared interoperable CDS service for the DAI check. The CDS service has been deployed on G-Cloud, and all authorized health care providers can use the service simultaneously through RESTful APIs. As of December 2021, 67 health care providers have participated in the initiative. Since the service developed in this study conforms with the CDS Hooks specification, clinical knowledge bases can be shared, and the services can be scalable.

According to the CDS service results report, the rate of warnings that occurred among the CDS service was 7.74% from 29 of the 67 participating hospitals for 1610 patients from May to August 2021. Among the warnings, the most frequent was the cross-reactive allergen check (43.55%), followed by the same drug or ingredient class check (28.77%) and the same product or ingredient check (27.68%). After warning responses from the CDS service, 9.07% of prescriptions were changed, and 90.93% were not changed [33]. Although warning responses occurred from the CDS service, physicians did not change their prescriptions, which had a rate of 90.93%. This proportion is similar to the range of average override alerts, 46.2% to 96.2% [34-36]. To induce physicians to change their prescriptions, additional information and services such as statistical data, research papers, or the SMART application could be provided as evidence.

We designed the CDS system based on serverless FHIR architecture. A CDS service can request additional data regarding the clinical workflow context to the FHIR server at providers via the hook parameters in the CDS Hooks specification. In Korea, the adoption of FHIR standards is in its infancy, and few health care providers have FHIR servers for requesting any additional data. Thus, we applied serverless FHIR architecture, identified the required data in advance, and assigned it in the prefetch field.

As awareness of national allergy codes increases, the MoHW of Korea is developing a national allergy code system. The KR Core AllergyIntolerance profile binds the KR Core AllergyIntolerance Code value set, a renamed version of the AllergyIntolerance Substance value set defined in the FHIR R4. The binding strength of the two value sets is preferred. It is meant to encourage drawing from the specified codes, but it is not required. Currently, there is no national allergy code system available in Korea, and the KR Core AllergyIntolerance Code value set is basically a placeholder for future value set development. Due to the lack of a national allergy code system, we chose to use a proprietary value set. When the national allergy code system is developed, it will replace the value set to draw from the national allergy code system with binding strength required, as well as the KR Core AllergyIntolerance Code value set.

### Conclusions

The shared interoperable CDS service for the DAI check based on the CDS Hooks was developed and deployed. The CDS service is currently provided to 67 health care providers. The MoHW has been making efforts to build the HL7 FHIR-based ecosystem in Korea. As one of these efforts, the CDS service initiative was conducted. To promote the rapid adoption of the HL7 FHIR standards, it is necessary to accelerate the practical service development and appeal the benefits of FHIR-based standardization to policy makers; this is the primary purpose of guiding the CDS service. Lastly, with the development of various case-specific IGs based on the KR Core IG, the FHIR standards will be distributed to the health IT industry, and more shared interoperable health care services will be introduced in Korea.

## Abbreviations

API: application programming interface
CDS: clinical decision support
DAI: drug-allergy interaction
DUR: drug utilization review
EHR: electronic health record
FHIR: Fast Healthcare Interoperability Resources
HIRA: Health Insurance Review and Assessment Service
HL7: Health Level Seven
IG: implementation guide
KD: Korea Drug
KR: Korea
MoHW: Ministry of Health and Welfare
RESTful: representational state transfer
SMART: Substitutable Medical Applications, Reusable Technologies
